# Chit-Chat

**Published:** 1874-10

**Authors:** 


### Editorial Chit-Chat.

The County Fair.—It was not remarkably successful. The exhibition was good, but attracted but few visitors, owing, probably, to lack of sufficient advertising.

Later.—We take it all back, as the attendance was large the third day.

Wanted.—We want a woman to do cooking; a middle aged, American woman, accustomed to plain cooking and bread-making. A woman who is able and willing to work, can find a good and permanent situation, and fair pay, by applying at the Surgical Institute.

Read It.—One of the very best articles we have ever read upon chronic invalidism, appears in this number of the Bistoury, from the pen of Dr. Hayes. No physician can read it without being impressed with its truthfulness, and no "chronic invalid" should fail to accept and profit by the gentle hints therein contained.

New Physician.—Our city is blest, or otherwise, with a large number of physicians, and still another is to be added to the list, this time, however, a very capable and experienced one, in the person of Dr. Doty, who comes from Canandaigua, to locate in the fifth ward of this city. We have met Dr. Doty a number of times in consultation, and know him to be a very superior physician.

Old Subjects.—Col. James Tripp, aged 83, of Dryden, N. Y., and Daniel Sherwood, Esq., aged 81, of Van Ettenville, N. Y., both soldiers in the war of 1812, and now hale and hearty, are among the inmates of the Institute. Col. Tripp has been blind for ten years, from *cataract*, and since the operation which restored him to vision, he feels as jolly and happy as a boy.

Mr. Sherwood has been blind about four years from the same cause, and is awaiting his turn for a similar operation. It is a rare entertainment to hear these old men relate their experiences of years ago, both having lived under every President from Geo. Washington to the present time. One proves to be a democrat and the other a republican, and political discussion is likely to wax hot between them. They seem to have lost none of their old interest or enthusiasm in matters relating to the State and Nation. "May they live long and prosper."

Don't.—Dr. Geo. H. Shrady, editor of the *Medical Record*, and Dr. Wm. A. Hammond, editor of the Psychological and Medico-Legal Journal, are making faces at each other through the columns of their respective journals. The latter has sued the former for slander; and it is all about mad-dogs—don't.

A Blessing After All.—It has been discovered that the potato bug, which has given us so much alarm, possesses qualities similar to those of the "spanish fly," and that the prospects are, instead of their being an unmitigated pest, they are about to become an article of real profit. A chemical manufacturing firm of Indianapolis, Ind., have given an order for one thousand pounds of the insects.

Olden Time Surgery.—We have been led to believe that extirpation of the eye-ball, as practiced of late years, (in cases of badly diseased eyes, where all hope of vision is lost and the diseased eye only remains to annoy the well one,) is of recent origin. Not so, however—for we are advised in the Bible, "if thy eye offend thee, pluck it out," showing the old patriarchs to have been "well up" in the requirements of ophthalmic science, and their advice is followed by all skillful oculists to the present day.

Swallowing a Tool-chest.—Rather a large undertaking, that,—but it has been successfully accomplished in some of the Paris prisons; at least we are so certified by a correspondent of the New York Medical Journal. It is stated that five or six deaths occur every year, in the prisons, from swallowing what is called the "escape-box." This remarkable box is made especially for the accommodation of such unlucky rascals as are "caught" and compelled to serve their time within prison walls. The box is of polished steel, about three inches long, and contains turnscrews, hammers, saws, silk thread, and other implements necessary for effecting an escape. The box is easily swallowed, but not always so easily passed,—"aye, there's the rub." When it fails to appear, as intended, the death of the victim is the result. But, upon the other hand, when it does pass the bowels, the lucky prisoner is prepared with a first-class set of tools to cut the thickest iron bars, and so effect his escape.

A Granger.—A gentleman residing in the lower portion of our city recently ordered and had sown *twenty-nine and one-half bushels* of grass seed upon less than three acres of ground! This reads like a joke, but it is not,—we saw the bill rendered, and it was $118. We recommend this gentleman as a very suitable candidate for the Grangers.

---

Quack Medicines.—Mr. Bidwell's article upon Patent Medicines, will lose none of its significance from the circumstance of his being an enterprising druggist in this city, and given largely to the sale of such nostrums. It is not often the case that dealers will say, as does Mr. Bidwell, "I keep patent medicines for those who *will* have them, but I charge you beforehand, most of them are worthless as remedial agents."

---

Inconvenience of Deafness.—The following is told by an exchange: There is a story of a country clergymen who was sent for suddenly to a cottage, where he found a man in bed. "Well, my friend," said the pastor, "what induced you to send for me?" The patient, who was rather deaf, appealed to his wife: "What did he say?" "He says," shouted the woman, "what *the deuce* did you send for him for?"

---

The Meat Question.—The President of the Massachusett's Society for prevention of cruelty to animals made an address to the society recently, from which we make the following extract, as being somewhat suggestive as to the character of meat city people are oftentimes compelled to consume:—

"It is estimated that about six per cent. of cattle, and about nine per cent. of sheep and swine, nearly 600,000 in all, annually die on the passage to market from the west, and a large portion of these are sold in our markets, either as meat, or rendered into cooking lard; while the cattle that get through alive, for the want of food and water, and by reason of the cruelty inflicted upon them, after losing on the average, in transportation, nearly a hundred pounds each in weight, from the most juicy and nutritious parts of the meat, come out of the cars full of fever, and with many bruises, sores and ulcers; and these, together with smaller animals, to which the loss and suffering is, in proportion, equally great, are sold in our markets for food."

The Liquor Dealers.--We have received an invitation to be present at the "Session of the New York State Convention of Liquor Dealers," to be held at Albany on Sept. 30th.

What is the matter with the liquor dealers? Something has frightened them, evidently. Is it the women's movement? If so, it is the first instance within our recollection of any "movement" looking toward a suppression of the liquor traffic, that has caused any alarm among the dealers.

---

Why Not?—Cannot we have a change of name for the principal street of the 5th Ward? It occurs to us that too many points of the compass are involved in the present name, which renders its exact location puzzling to strangers. Having one Water St., "South Water St.," is often taken for the lower portion of Water St., and when we add "West South Water St." the matter becomes perplexing. Let us have a new name, therefore, for South Water St. Call it a "Place," as it is a very short street,—name it "Wyckoff Place," for instance, in honor of the gentleman who has done so much toward beautifying it. At all events give us a change of name.

---

That Infernal Noise.—Is there any earthly necessity for those abominable shrieking, skreeching, whistles that are given mostly by the Northern Central Railway engines, as they leave the bridge, going south? It may be fun for the engineers, but it is next to death for the inhabitants of the 5th Ward, especially those residing within a mile of the track. We have known sick persons to be painfully disturbed by the terrific whistles referred to, and we think if the fact could be known to the managers of the road, they would adopt some less noisy system of signaling.

Massachusetts has passed a law absolutely forbidding the use of steam whistles of any sort being used within her borders. Have we not endured this intolerable nuisance long enough? Is not the list of *murdered* citizens, broken vehicles, ruined horses, and broken bones sufficiently long to warrant the people in offering a strong protest to the further tolerance of steam whistles? Let us have them legislated out of existence, for other means of signaling, quite as efficient and divested of noise and consequent danger and annoyance, are at hand.

From "H."—This is his latest, taken upon the wing, during his recent vacation in search of woodcock: Some time ago an inmate of a certain lunatic asylum disappeared rather mysteriously, and after the lapse of several months, his remains, reduced to a skeleton, were discovered, and the coroner held his inquest as usual. A few days ago the man was found living on Deep Island, Boston Harbor, much to the disgust of the aforementioned coroner, who declares that no one but a mad man would go off and leave his skeleton behind him.

---

Splendid Organ.—One of the finest organs we have ever seen or listened to, is the one in the First Methodist Church of this city. We were induced to go and hear it by those who are competent judges of the good points of instruments of this kind, and could but agree with them, that it is a very superior instrument, both in tone and finish. We learn the instrument was manufactured in this city, by Mr. W. King, organ builder, on Church St. We see no necessity for religious societies paying the necessarily larger sum for organs purchased in New York and Boston, when better instruments can be had in Elmira.

---

Didn't Pay.—We hear of an individual in this city who thought he would save a tooth-pulling fee, by attempting the operation for himself. Mindful of his boyhood experiences, he tied a stout "waxed-end" securely to the offending inscisor, securing the other end to a smoothing iron, he mounted a chair, thinking if he would allow the iron to fall, the sudden jerk would remove the tooth so quickly as to leave no pain in its wake. The thought was a brilliant one, but miserably failed in its execution, for, when everything was in readiness, he closed both eyes, let go the iron, when it fell, not to the floor, but upon his favorite corn, causing such an astonishing number of curious and exceedingly nimble gymnastic feats as to amaze a choice number of spectators who witnessed the entertainment. It is true, the tooth was removed, together with an inch or so of the gum, and all without pain—to the tooth—but the circumstance of the operator now appearing upon our streets with a home-made pair of crutches, leads us to the belief that the experiment was not so successful as was hoped for.

Dental Bill.—Atkinson, a New York dentist, has brought suit against the celebrated actress, Agnes Ethel, to recover a bill of $1,025.00 for teeth filling. She says the bill is exorbitant. It certainly looks as though Agnes was right about it.

---

Japanese Medical Journal.—The Japanese are evidently progressing. Dr. Stuart Eldridge, an American physician connected with the staff of five large native hospitals on the island of Yesso, Japan, has a large number of native students under instruction, and is publishing a bi-monthly illustrated medical journal, in the Japanese language.

---

Annoyance.—The indications are that the "Music Hall" located in the Opera House block, will prove an intolerable nuisance. It was a brilliant conception of the proprietors of the Opera House property, when they disposed of one of their stores to be used as a musical lager bier saloon; the patrons of the Opera House can now be entertained by two musical entertainments for the price of one, a clear gain of one concert. Recent experience however leads us to the decision that one concert at a time is much preferable, and we believe our opinion is shared in by a large portion of the public who have recently visited the Opera House.

---

Just Think of It.—Dr. Drysdale, an eminent English physician, who has given much of his time to investigating the effects of tobacco upon the system, takes strong grounds against its use. In a paper read at the annual meeting of the British Medical Association, he affirmed that within one week he had seen two young men affected with almost complete blindness, induced in one case, by excessive smoking, and in the other, by chewing tobacco. He relates other cases of disease superinduced by the use of tobacco, such as dyspepsia — with intermittent pulse, diarrhœa, piles, palpitation of the heart, and functional diseases of that organ, paralysis, etc. In our own experience, we almost daily come in contact with diseases of the eye and respiratory organs, which can be directly traced to the use of tobacco. The use of this weed is becoming so extensive that the injurious consequences arising from it, will scarcely be surpassed by that of alcoholic drinks.

Needs Protection.—Woodlawn cemetery is invaded by a set of thieves too contemptibly mean and degraded to be called human beings. Articles left upon the graves, as a means of beautifying them, such as boquets, vases, baskets, and such ornaments as can be carried, are ruthlessly abstracted and stolen. Watering-cans and garden implements used in keeping the grounds in order are also carried away by these human vampires. As it is impossible for Mr. Abbott to patrol the grounds and attend to his duties as sexton, should not a special policeman be appointed for these grounds during the summer months? We are sure much more would be done by lot owners toward embelishing their grounds with flowers and vases could they be assured of their proper protection.

---

Try It.—Take a stereoscope, such as is ordinarily used for examining stereoscopic pictures, and is found now-a-days, on almost every parlor table, and amuse yourself with the following experiment, which is one of the means employed by the ophthalmic surgeon in detecting pretended blindness: Mark on the back of one of your pictures, in good sized letters, "I See." Now, if both eyes are used in reading this through the stereoscope, it will appear as "See I," a transposition of the sentence and of its meaning. If *one* eye only is used, or if one eye is blind, the sentence will read as written, "I See." If without removing the instrument from the eyes you attempt to draw a perpendicular line between the words thus: "See | I," you will be surprised upon removing it to find that your mark will appear thus—I see | .

---

Dusting.—Our medical exchanges have much to say expostulatory of the views expressed by M. Gosselin, relative to the dusting of sick wards during their occupancy by patients. M. Gosselin asserts that much of the fatality occuring in the large Parisian hospitals, is the result of "too much cleanliness," in the wards. He says to the sisters: "You are always scrubbing the floors, sweeping down the walls, brushing off the curtains, and shaking the bed-clothes. Now, all persons are agreed that this dust which you are incessantly removing, is loaded with zymotic germs. *Let it lie*, therefore. So long as it reposes in peace it is harmless; when you fill the atmosphere with it, it flies hither and thither, settles upon the wounds, touches upon the mucous membranes, and enters into the system of the patients by the mouth, nostrils, the air-tubes and the digestive passages." We see nothing very improbable or absurd about this proposition; contrariwise, we deem it a very sensible view of the situation. We doubt not that much of the fatality attending surgical operations in the large hospitals, is directly attributable to poisoning by zymotic germs deposited upon wounds, in the manner described by M. Gosselin.

---

Tapeworm in Grouse.—A writer to the *Lancet* says: "Having read in some late numbers of your journal letters on the grouse disease, I was induced to examine the intestines of as many grouse as I could obtain, with the view of ascertaining whether they were generally affected with tapeworm or not. I have now examined the intestines of seventy-two birds, in all of which I have found tapeworm, with the exception of eight. It must be borne in mind that the birds which I examined were not diseased birds, in the common acceptation of the word, but marketable birds, which were eaten. In some few cases I found the intestines perforated by the worms, but of course cannot say that it was done during life. In most cases the intestine was apparently entirely blocked up with them for a length varying from two to six inches. This occurred always about the same place in the intestine."

Years ago, while grouse shooting upon the Western prairies, our attention was called to the almost universal prevalence of tape worm in the intestines of the grouse. In view of the danger of the eggs of the parasite being deposited in the flesh of the bird, through a perforated intestine, it would be well to have such game birds thoroughly cooked. Such a process would effectually destroy any stray eggs that might otherwise find lodgment in the human intestinal canal.

---

Our Barometer—it's a goood one—was given us by a friend who had used it with success for a number of years. He kept it in his bank, and assured us that the rise and fall of the money market was correctly indicated by it. So constantly was he reminded of this fluctation that he became nervous and as uncertain as was the price of gold, consequently transferred it to his dwelling and from thence into our hands. Since then, we have watched it daily, and have been delighted with its wonderful accuracy. When it rains, the barometer indicates the fact,—after the shower has fairly set in, so there can be no mistake about it, for in its downright truthfulness consists its value. When the rain ceases, the barometer is on hand to indicate a change, which it does with the utmost promptitude and seeming cheerfulness. We have noted for a number of years, that a storm is usually followed by a calm, and would you believe it, that confounded barometer has discovered that, also! We have seen many barometers in our day, have watched them, and then guessed with varying degree of accuracy as to what would happen next, but this instrument of Frank's, is the most startling in its prognostications of any we have ever examined. You see, it is so confoundedly particular to be absolutely correct and truthful, that it often waits an hour or two after a rain has commenced, before it will tell you to prepare for a shower. To persons who are confined much to indoor work, and who can rarely get into the open air—criminals and lunatics, for instance, this barometer of ours is invaluable. But for those who can occasionally look out at the window, the indications come too late to be of any appreciable value.

---

WHEW.—We, in common with housekeepers in general, are constantly annoyed with incompetent help. Individuals from a class of community upon whom we are in the habit of relying for aid in carrying on the drudgery of household work, have proved so inefficient and worthless, as to cause people to experiment with foreign help, taken from the southern negroes, the Germans, and recently the Chinese. We have heard many good reports from those who have employed Chinese labor, —have heard of their patience, honesty, industry, aptitude in imitation ; quickly learning what is taught them, and above all, working for *small wages.* Having all these excellencies in view, and being extremely desirous of securing capable and reliable help in the Institute, we addressed a letter of inquiry to a friend in San Francisco, Cal., asking information upon the following points :—

Can Chinese men be induced to come east in squads of ten or less ?

Are the men easily taught, and do they make good cooks, "chamber-men" and laundrymen ?

Are they trustworthy, and what wages do they demand ? And here is the reply. It suited us exactly, excepting in the matter of wages. The idea of paying a man sixty to seventy-five dollars per month for doing plain cooking, and in addition, defraying his transportation expenses, amounting to $120 more, is placing a higher estimate upon such services than we are accustomed to considering in this part of the world. But here is an extract from our friend's letter :

"I think you cannot do better than to get Chinese servants for your Institute. I know all about them. They are patient, steady, industrious and civil, when not abused, but when insulted are revengeful and malicious. I have tried them with Irish servants, and would rather have them, *ten to one.* There is no comparison at all.

"I can get a cook for you for $60 a month, if the number of boarders does not exceed thirty. If less, I can get one for less, and if more, I will have to give more. Also, if you want an excellent cook, one of high attainments, I can get one for $75, or a plain cook for a small number of people for $30. You can take your choice. A chamber man will cost you $30 and a laundryman $35 to $40, depending on quality.

"I will interest myself to get you good servants, with good recommendations ; and, indeed, what I tell you now about prices, &c., is the result of considerable inquiry. They do not like to venture alone into the centre of the continent, and it will require some trouble to 'carrol' them.

"If you know two or three or more people that want servants, it will be easier to make up a gang of ten or a dozen than to get three men alone. An order for a dozen men can, I think, be easily filled ; an order for three men will not be.

"It will be necessary to send about $120 for each man's expenses there—second cabin, and the middle man's commissions.

"Let me know then what you want, how much you will pay, and what I must do, and I will do all I can to help you get here what you never can get there, a lot of real good servants."

Nevertheless, we often could be induced, and doubt not many of our readers are likewise inclined, to pay twice the wages we are accustomed to paying, could we but get rid of the annoyance of constantly looking for "new help." We publish so much of our friend's letter as relates to the help question, thinking it may not prove uninteresting to many who would like to secure servants even upon the terms mentioned.

